# Are Sports-Related Factors Correlated to the Prevalence and Initiation of Illicit Drug Misuse in Adolescence? Prospective Study in Older Adolescents

**DOI:** 10.1155/2018/1236284

**Published:** 2018-11-28

**Authors:** Zoran Zubak, Admir Terzic, Natasa Zenic, Ljerka Ostojic, Ivana Zubak, Mario Jelicic, Haris Pojskic

**Affiliations:** ^1^Special Hospital Biograd, Biograd 23210, Croatia; ^2^Faculty of Medicine, University of Mostar, Mostar 88000, Bosnia and Herzegovina; ^3^University of Tuzla, Tuzla 75000, Bosnia and Herzegovina; ^4^Faculty of Kinesiology, University of Split, Split 21000, Croatia; ^5^Academy of Medical Sciences, Sarajevo 71000, Bosnia and Herzegovina; ^6^University of Zadar, Zadar 23000, Croatia; ^7^Linnaeus University, Department for Sports Science, Kalmar 39182, Sweden; ^8^Swedish Winter Sports Research Centre, Mid Sweden University, Östersund 83125, Sweden

## Abstract

Sport participation is considered as a factor of potential influence on illicit drug misuse (IDM) in adolescence, but there is an evident lack of studies which prospectively investigated this problem. This study aimed to prospectively investigate the sports-related factors related to IDM and the initiation of IDM among older adolescents. The participants were 436 adolescents (202 females; 16 years old at study baseline). They were tested at baseline and follow-up (two years later). The predictors included variables associated with different facets of sports participation and success in sports. The criteria were (i) baseline IDM, (ii) follow-up IDM, and (iii) initiation of IDM between baseline and follow-up. Crude and adjusted (controlled for parental conflict, age, socioeconomic status, and gender) logistic regressions were applied to establish correlations between predictors and criteria. There were higher odds for baseline IDM in adolescents who quit individual sports (OR: 4.2, 95% CI: 1.3-13.9), who had better competitive sports achievements (OR: 1.8, 95% CI: 1.0-3.3), and those involved in sports for a longer time (OR: 1.6, 95% CI: 1.0-2.5). The IDM at follow-up was more prevalent in adolescents who were involved in sports for a longer time (OR: 1.7, 95% CI: 1.1-2.6). Initiation of drug use was predicted by longer experience in sports (OR: 1.8, 95% CI: 1.1-3.1). Sports-related factors were more negatively than positively related to illicit drug use. Most probably, the transition from junior to senior level in sports put specific stress on those adolescents who were highly committed to sports until that time, but who then had to question their own sports abilities and future potential in sports. Sport-authorities should be informed on established results and specific public-health efforts aimed at preventing IDM in athletic adolescents are urgently needed.

## 1. Introduction

Consumption of illicit drugs (i.e., heroin, LSD, ecstasy, amphetamines, cocaine, crack, amphetamines, hashish, barbiturates, codeine, cannabis, and GHB) is a serious health threatening behavior, connected with numerous detrimental health-effects [1–3]. Also, illicit drug misuse (IDM) is related to serious social problems as well [1, 4]. The drug users suffer harmful consequences of drug addiction, which generates an enormous medical, financial, social, and emotional burden on individuals, their families, and our society [5]. Although most teens do not escalate from so-called “trying drugs” to developing addictive behaviors, even experimenting with illicit drugs is a problem. Namely, even irregular IDM can result in serious consequences (i.e., unsafe sex, intoxicated driving, injecting, and violent behavior), and therefore even the rare IDM can pose a serious health-related (i.e., HIV, hepatitis-C) and social-related (i.e., traffic accidents, violence) risk [6–8]. Although serious social- and health-related problems are related to consumption of other substances and not solely to IDM, the fact is that the consumption of other substances (i.e., alcohol and tobacco products) is at least partially legal in most countries. At the same time, the IDM is regularly considered illegal behavior. From the view point of preventive efforts, education, and public health, this is very important, since IDM places those who misuse drugs in criminal milieu [9, 10].

There is a wide consensus that special effort is needed in order to develop effective preventive campaigns against illicit drug use in adolescents. One of the possible approaches is identification of the precipitating factors of IDM [11–15]. Briefly, the idea is (i) to find those factors that are associated with consumption of illegal drugs, (ii) identifying these factors will allow targeting of those adolescents who are at specific risk for usage of illicit drugs and consequently, and (iii) it will assure the development of accurate and specifically tailored preventive programs. Among those factors that are potentially related to substance misuse in adolescents are variables of sports participation [16]. For example, one of the most effective preventive-models against substance misuse in adolescence was developed and successfully applied in Iceland [17, 18] and sport participation appeared to be one of the most important factors in model-development [18].

Because of the physical exercising in sports, the sport participation in youth age results in positive effects on physiological status, with proven effects on cardiovascular-status, motor-qualities (i.e., strength, flexibility, balance), and anthropometric/body build indices (i.e., prevention of overweight and obesity) [19–21]. Additionally, psychosocial benefits of sports participation are also frequently reported [19, 22]. Not surprisingly, the role of sports participation in the prevention of IDM among adolescents is repeatedly observed [11, 23, 24]. Although anecdotally sports may be considered to be “protective” against IDM, results of previous studies did not confirm such theories. Briefly, some studies reported protective effects of sports and found lower prevalence of IDM in athletic adolescents than in their nonathletic peers [25–27]. However, investigations did not regularly found the protective effects of sports against the IDM, and in some reports athletic adolescents were more likely to consume illicit drugs than those who were not involved in sport-programs [11, 28]. Collectively, it seems that connection between sport participation and IDM should be explored specifically for different communities, cultures, and regions, while paying attention on different facets and variables of sport involvement (i.e., individual versus team-sport, current-former-never participation, competitive achievement) [29].

Recent cross-sectional studies done on the territory of former Yugoslavia, including Bosnia and Herzegovina (B&H), have identified alarming figures about substance misuse in adolescents [30–32]. Also, previous studies investigated different groups of factors that are potentially related to cigarette smoking, alcohol consumption, and the IDM in this country and territory. Among other factors (i.e., parental, scholastic, and socioeconomic factors) investigations reported associations between sport-related factors and substance misuse, including IDM [29, 33, 34]. While some sport factors were found to be negatively associated with the IDM in adolescents, others were found to be positively related [29, 33, 34]. Specifically, sport participation was not related to IDM in 18-year old adolescents [35], but when sport factors were observed more specifically (current-former-never participation), those girls who quit sports were at high risk for IDM [29]. Also, results indicated specific associations between sport-success and IDM, with higher risk for IDM in those adolescents who competed in sports but achieved poor competitive result [29].

Apart from their important findings, the previously cited studies had certain limitations [29, 35]. Most specifically, the cross-sectional design evidenced the associations but did not allow interpretation of the causality between observed variables. Indeed, while some associations may be intuitively interpreted, the cross-sectional approach did not allow more accurate identification of the cause-effect relationships between sport factors and substance misuse [29, 35]. This problem is additionally highlighted in more recent studies where authors prospectively investigated different predictors of substance use and misuse in adolescence and clearly evidenced causality between variables [6, 36, 37]. Therefore, this study aimed to prospectively investigate the relationship between sport factors (predictors) and the IDM (outcome) in 16- to 18-year-old adolescents from B&H. Apart from evidencing the associations between the predictors and the outcome at study baseline (at the age of 16) and follow-up (at the age of 18), we aimed to evaluate the influence of the observed predictors on the initiation of IDM in the observed period (i.e., between 16 and 18 years of age).

## 2. Materials and Methods

### 2.1. Procedures and Participants

**Figure 1:**
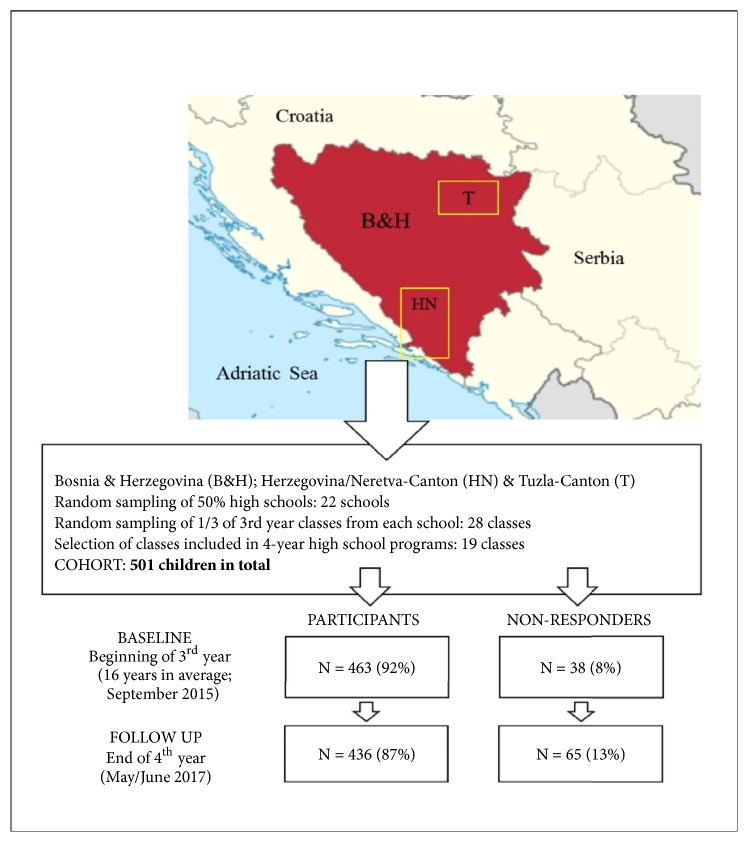
Location of the study, sampling, the drop-out, and retention rates.

The sample of participants comprised adolescents from Bosnia and Herzegovina, who were 16 years old and attended 3rd year of high school at study baseline. They were prospectively observed during the next two years, and were again tested at the end of 4th year of high school (follow-up), when they were 18 years old in average (Figure 1). Originally, the sample of participants consisted of 501 children, but we observed only those adolescents who were tested at baseline and follow-up (n= 436; 46% females). On the basis of data on the prevalence IDM from a previous cross-sectional studies performed in Bosnia and Herzegovina of 10% [34, 35], with population/theoretical sample of 8915 children and level of significance of p < 0.05, the required sample size was 137 participants. For the purposes of selecting participants, we used a multistage simple random sampling method. The sampling, drop-out, and retention rates are presented in Figure 1.

Sampling was based on a multistage simple random sampling method. At the first stage, all high schools Tuzla Canton and Herzegovina-Neretva Canton were sorted into two groups according to number of pupils. Next, we randomly selected 30% of 3rd year classes from each group and additionally selected only classes which were involved in four-year high school program (e.g., some high school programs in the country finish with 3rd year). At the final stage, one-school shift was randomly selected. This process altogether resulted in a cohort of 501 children.

The study was anonymous, and no personal data were gathered. Meanwhile, in order to follow responses between baseline and follow-up, participants were asked to use a self-selected confidential code. To assure that participants will remember selected code across testing waves, they were suggested to use last three digits of the e-mail password. Ethical approval was obtained from the Ethical Board of the corresponding author's institution (Ethical Approval No: 2181-205-02-05-14-005). During the first week of the school year, at the regular school meeting, the study design and aims of the investigation were presented to parents/guardians of the children, who gave written consent for participation in the study. Testing was organized during school hours and took approximately 15 minutes. When testing was done, participants sealed the questionnaire form and put it in a closed box, which was open next day by a n investigator who did not tested participants. Baseline and follow-up testing were done only once, and consequently study did not include adolescents who were not at school on a testing day. The same protocol of sampling and testing was followed in previous cross-sectional studies that have examined the problem of substance use and misuse in adolescents from the territory [11, 29, 34, 35], while one very recent study prospectively examined the same sample of participants in order to evidence the relationship between scholastic variables and illicit drug misuse [6]. Therefore, authors are of the opinion that the results obtained herein may be objectively compared with those previously reported [6, 11, 29, 34, 35].

The attrition bias analysis (Supplementary table (available here)) showed no significant differences in the initial consumption of illicit drugs between those adolescents who remained in the study and those who dropped out (Chi square: 2.51, p = 0.11). There were significantly more males than females who dropped out (Chi square: 6.17, p = 0.01). The intracluster correlation coefficient calculated for baseline smoking prevalence, with the individual schools as clusters, was 0.06, indicating appropriate within-cluster (i.e., within-school) variance [38, 39]

### 2.2. Variables

The predictors were sport-related factors (sport factors). The outcome was the IDM. On the basis of previous studies in which investigators repeatedly reported associations between gender, parental conflict, socioeconomic status, and age, these variables were included as covariates. Question on conflict with parents (parental conflict) included four possible answers (never, rarely, from time to time, frequently), while socioeconomic status was tested on three-point scale (under average, average, and above average). The previously validated questionnaire (Questionnaire of Substance Use, QSU) was used for testing and questionnaires were provided in local language [34, 35].

Questions on sport factors included questions on the following: (i) involvement in competitive individual sports; (ii) involvement in competitive team sports (both answered on a scale consisting of the following three possible answers: never involved, quit, or currently involved); (iii) highest achieved competitive sports result (never involved/competed, competed locally, or competed nationally/internationally); (iv) duration of sport involvement (never participated, participated for less than a year, participated for 2-5 years, or participated for more than 5 years); and (v) number of training sessions per week (for those who participated in sports; four point scale from “1-2 per week” to “sometimes even twice a day”). The individual- and team-sport participation were studied independently, since previous studies identified possible differential influence of these two groups of sports on substance misuse patterns in adolescence [40, 41]. Also, because of the great body of evidence that suggests that the IDM may vary by current (and not overall) level of experience in sports [11, 29], in this study, sports participation was evaluated on a scale where participants were not simply asked “are you participating in sports or not?” (i.e., a yes-no scale) but the possible answers included that they had “quit” sports.

The outcome variable was the consumption of illicit drugs. The scale for drug consumption included questions about the consumption of marijuana, hashish, heroin, cocaine, sedatives, and most party drugs (e.g., ecstasy, amphetamines). Participants were later categorized as “drug users” (those who ever tried any of the illicit drugs) and “nonusers” [34].

### 2.3. Statistics

The counts (frequencies) and percentages were reported for all variables. The Mann-Whitney* U* test (MW) or the Chi square test was calculated to establish the differences between drug misusers and nonusers. The relationships between sport factors (predictors) and the IDM and the logistic regressions were calculated. The criteria were (i) IDM at baseline; (ii) IDM at follow-up; and (iii) the initiation of IDM between testing waves. The crude (nonadjusted) logistic regression analysis, and adjusted logistic regression model (adjusted for age, gender, socioeconomic status, and conflict with parents) were calculated with odds ratio (OR), and corresponding 95% confidence intervals (95% CI) reported. The logistic regression calculated for the initiation of IDM did not include participants who were identified as drug misusers at study baseline. Statistical significance of 95% was applied and Statistica ver. 12.0 (Statsoft, Tulsa, OK) was used for statistical calculations.

## 3. Results

**Figure 2:**
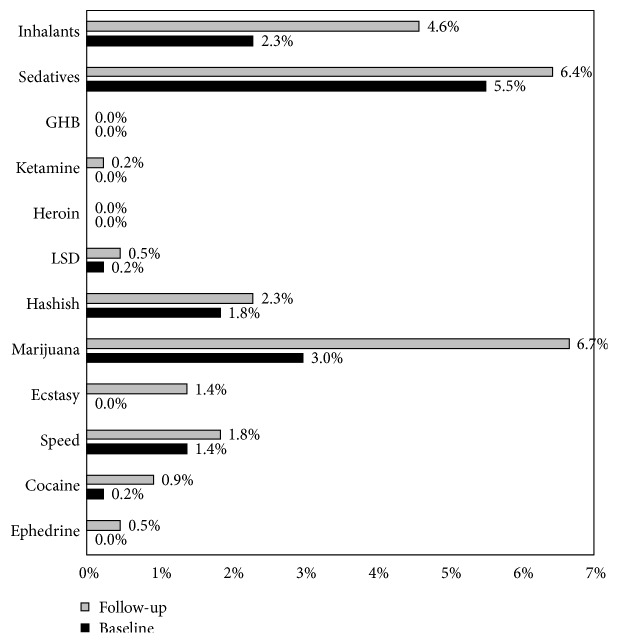
Prevalence of misuse for different drugs at study baseline and follow-up.

Overall, 5% of adolescents were identified as illicit drug misusers at the beginning of their 3rd year of high school, and 7% misused illicit drugs at the end of high school 20 months later. Distribution of misuse for different drugs is presented in Figure 2.

Table 1 presents the distribution of independent variables according to illicit drug use status at baseline and follow-up. Sport factors did not differ drug misusers and nonusers at study baseline. Those who misused illicit drugs at follow-up were involved in sports for a longer time (MW: 2.93, p<0.01).

Quitting individual sports (OR: 4.22, 955% CI: 1.28-13.94), better competitive result achieved in sports (OR: 1.84, 95% CI: 1.02-3.32), and longer experience in sports (OR: 1.60, 95% CI: 1.01-2.53) were factors of increased risk for IDM at study baseline. Also, longer involvement in sport was correlated to IDM at follow-up (OR: 1.70, 95% CI: 1.12-2.58) and initiation of IDM during the course of the investigation (OR: 1.78, 95% CI: 1.02-3.12) (Table 2).

## 4. Discussion

There are several main findings of this study. Quitting individual sports, better competitive achievement, and longer involvement in sports were shown to be specific risks for IDM. Those who were involved in sports for a longer time were at risk for the initiation of IDM.

### 4.1. Association between Sport Participation and Illicit Drug Misuse in Adolescents

The positive influence of practicing sports during childhood and adolescence is related to both the positive effects on physiological status due to physical exercise and the psychosocial benefits of sports participation [25]. Not surprisingly, one area that has been receiving attention is the role of sports participation in the prevention of IDM among adolescents [11, 23, 24]. Our results showed specific negative associations between sport factors and the IDM. In brief, quitting sports and higher sports result (competitive results achieved in sports) were found to be associated with a higher likelihood of IDM at baseline. Additionally, a higher risk for IDM at follow-up was found in those adolescents who reported longer involvement in sports.

Although anecdotally sports may be exclusively considered to be “protective” against IDM, our results on the negative effect of sports participation on the consumption of IDM are not as surprising as they may appear to be at first glance since some previous investigations reported similar findings [11, 28]. Evidently, the group of former athletes is recognized as susceptible to the IDM at the age of 16. The question that arises is, did they (i.e., former athletes) start to misuse illicit drugs before or after quitting sports? Further discussion may help to find a path for a profound interpretation of this issue.

At the age of 18 years (i.e., the time of the follow-up measurement), longer sports participation was identified as a risk factor for the IDM. Comparing the correlations obtained at baseline and follow-up measurements, it seems obvious that those who were continuously involved in sports started to consume illicit drugs “later” than nonathletes and “later” than those who quit sports before the age of 16. Indeed, even the frequency tables confirm this conclusion. It is important to note that when evaluating the whole course of the study, nonathletes (i.e., those who were never involved in sports) were evidently at a lower risk for IDM than their peers who participated in sports at any level.

This finding on higher IDM in athletic adolescents is consistent with a recent report from a cross-sectional study on this problem in adolescents from Kosovo [11]. In that study, the authors performed a gender-specific analysis and found a high risk for illicit drug usage in athletic girls (girls who reported better sports success were more prone to IDM). This association was explained by the specific sociocultural environment of sports society in Kosovo. In brief, girls were involved in sports travel more often because of sports competitions and/or training. Consequently, they are more often in “out-of-home” situations and are consequently in a “noncontrolled” environment, where they are more likely to start consuming drugs [11]. Knowing the similarities of the sociocultural milieu in B&H and Kosovo (mostly because of the former Yugoslav tradition and consequent similarities in cultural and social norms), this explanation is plausible for our study as well.

### 4.2. Influence of Sport Participation on Illicit Drug Misuse

Findings on cross-sectional associations between sport factors and IDM can be interpreted as each of the study variables as “the cause” [11, 34]. However, the prospective relationships identified in this study are clear. In short, experience in sports was a risk factor for the initiation of IDM during the studied period of time (i.e., between 16 and 18 years of age). It must be highlighted that this causality is a characteristic only for those adolescents who did not consume drugs at the study baseline (for more details please see the subsection on statistics). This finding actually confirms the previous explanation about the origin of the established association between “experience in sports” and IDM at the follow-up measurement.

Apart from the previously discussed problem of more frequent absence from home, which is common for athletes, there is another possible background factor leading to a higher risk of initiating IDM in more experienced athletes. Being actively involved in sports programs in the country, the authors of the study are well aware of the fact that, during the studied period of time (between 16 and 18 years of age), the most rigid selection process for sports occurs because this is the period of transition from a junior to a senior competitive level [42]. The majority of the youth athletes will quit sports at this age, mostly because there is no organized sports training (or competitions) for those who are not selected for senior level competitions (e.g., professional level). As a result, this is a highly stressful period of time for those involved in sports.

Consequently, drug initiation is more probable in those who practice sports for a prolonged period of time. Namely, those who are actively involved in sports for a prolonged time are highly committed to sports training (i.e., otherwise they would have already quit). At the same time, most of them can objectively predict that they will not be able to pass the following selection process [42, 43]. The frustration of those young individuals is clear and leads to higher likelihood of consuming drugs. Not surprisingly, the likelihood of drug initiation during studied time frame was significantly higher in athletic adolescents than in nonathletic adolescents, simply because nonathletic adolescents were not exposed to the same kind of stress as their athletic peers.

One can argue that the quantitative study design applied in this study does not allow unquestionable interpretation of this relationship, and this certainly holds true. Although we must not ignore the fact that more detailed qualitative analyses are needed, some previous studies indirectly confirm the previous discussion. Although we could not find any study which investigated the problem of failure in sport and consequent “frustration” as factors which could result in IDM in adolescent athletes, the findings of studies which investigated doping behavior (i.e., usage of the performance-enhancing-drugs) in sport are clearly supportive to previous discussion. Briefly, when observed athletes were involved in competitive sports, studies regularly confirmed higher likelihood of doping behavior (i.e., positive attitude toward doping) in those athletes who did not achieve high competitive results but were strongly committed to sports [42, 44, 45]. While “doping behavior” in sports is actually a type of “drug consumption”, the similarity between findings is clear.

### 4.3. Limitations and Strengths of the Study

The main limitation of this study is that it was based on self-reported data. Therefore, participants may have tended to provide socially acceptable answers. However, we believe that the study design and our experience from previous studies in the protection of the anonymity of the participants reduced this possibility. Also, one can argue that problem of religiosity/ethnicity was not sufficiently explored, especially knowing that significant part of population in Bosnia and Herzegovina are Muslims. However, previous studies done on adolescents in Bosnia and Herzegovina did not confirm differences in IDM between different ethnicities (i.e., religious affiliations), and therefore religiosity was not observed as a variable of potential influence on IDM herein [29]. Additionally, this investigation was quantitative, and some important qualitative data were missing for a more profound interpretation of the results, such as the reasons for quitting sports and self-evaluations of future involvement in sports. Finally, this study examined specific sample of adolescents from one country and therefore generalizability is limited.

This is one of the first studies conducted in southeastern Europe and probably the first one in the territory of former Yugoslavia to prospectively examine the association between sport variables and IDM in adolescents. The sport variables were investigated in detail, and prospective study design allowed not only the identification of the relationships but also the interpretation of the causality between the studied variables.

## 5. Conclusions

Our results suggest that quitting sports is a risk factor for the earlier initiation of IDM (i.e., before 16 years of age), but this should be systematically investigated in further studies with younger participants. Also, it seems that the transition from junior to senior level in sports put specific stress on those adolescents who were highly committed to sports until that time, but who then had to question their own sports abilities and future potential in sports. In developing effective and accurate preventive efforts against IDM, here evidenced factors associated with higher risk should be taken into account. Sports authorities should be informed of the present findings of higher risk for IDM in certain groups of former/current athletes, and specific efforts aimed at preventing IDM in athletic adolescents are urgently needed. Also, because of the high prevalence of consumption of sedatives, the background of usage (prescription/nonprescription drugs, specific types of used sedatives, etc.), as well as specific correlates of such behavior (frustration, familiar problems, scholastic failure, etc.), should be more precisely investigated in the future.

## Figures and Tables

**Table 1:** Sport factors at the baseline and follow-up with differences according to misuse of illicit drugs (MW, Mann Whitney Z values; Chi square test, Chi^2^).

	**Baseline**			**Follow-up**		
	**Nonusers**	**Misusers**	**MW**	**Nonusers**	**Misusers**	**MW**
	**F (**%**)**	**F (**%**)**	**Z (p)**	**F (**%**)**	**F (**%**)**	**Z (p)**
**Sport success/result**						
Never competed	226 (54.2)	6 (31.58)	-1.92 (0.06)	217 (53.32)	12 (41.38)	-1.36 (0.17)
Local rank	162 (38.85)	11 (57.89)		153 (37.59)	13 (44.83)	
National rank	26 (6.24)	1 (5.26)		28 (6.88)	3 (10.34)	
International rank	3 (0.72)	1 (5.26)		8 (1.97)	0 (0)	
Missing	0 (0)	0 (0)		1 (0.24)	1 (3.45)	

**Number of training sessions per week**						
1-2 per week	130 (31.18)	4 (21.05)	-0.91 (0.36)	144 (35.38)	10 (34.48)	-1.16 (0.23)
3-5 per week	151 (36.21)	8 (42.11)		130 (31.94)	12 (41.38)	
Every day	34 (8.15)	3 (15.79)		41 (10.07)	3 (10.34)	
Sometimes even 2 sessions per day	6 (1.44)	0 (0)		12 (2.95)	1 (3.45)	
Missing/not participated in sport	96 (23.02)	4 (21.05)		80 (19.65)	3 (10.34)	

**Experience in sport**						
Never been involved	114 (27.34)	4 (21.05)	-1.41 (0.15)	112 (27.52)	3 (10.34)	-2.93 (0.01)
< 1 year	91 (21.82)	3 (15.79)		82 (20.15)	5 (17.24)	
2-5 years	114 (27.34)	4 (21.05)		124 (30.47)	8 (27.59)	
> 5 years	98 (23.5)	8 (42.11)		88 (21.62)	12 (41.38)	
Missing	0 (0)	0 (0)		1 (0.24)	1 (3.45)	

**Individual sport participation**			**Chi** ^**2**^ ** (p)**			**Chi** ^**2**^ ** (p)**
Yes, still participating	84 (20.14)	8 (42.11)	5.13 (0.07)	74 (18.18)	5 (17.24)	0.67 (0.71)
Quit	138 (33.09)	5 (26.32)		152 (37.35)	13 (44.83)	
Never	195 (46.76)	6 (31.58)		181 (44.5)	11 (37.93)	
Missing	0 (0)	0 (0)		0 (0)	0 (0)	

**Team sport participation**						
Yes, still participating	94 (22.54)	6 (31.58)	1.77 (0.41)	80 (19.66)	5 (17.24)	1.01 (0.60)
Quit	171 (41.01)	5 (26.32)		172 (42.26)	15 (51.72)	
Never	152 (36.45)	8 (42.11)		155 (38.08)	9 (31.03)	
Missing	0 (0)	0 (0)		0 (0)	0 (0)	

**Table 2:** Correlates of illicit drug usage at baseline and follow-up, and initiation of illicit drug use during the study course.

	**Baseline**		**Follow-up**		**Initiation ** ¥	
	**OR**		**OR**		**OR**	
	**(95**%** CI)**		**(95**%** CI)**		**(95**%** CI)**	
	**Crude**	**Adjusted**	**Crude**	**Adjusted**	**Crude**	**Adjusted**
**Individual sport participation**						
Yes, still participating	1.18	1.26	1.10	1.07	4.89	3.99
	(0.35-3.94)	(0.37-4.35)	(0.37-3.29)	(0.33-3.46)	(1.19-20.07)	(0.90-17.68)
Quit	3.08	4.22	1.41	1.44	3.42	3.28
	(1.04-9.15)	(1.28-13.94)	(0.61-3.23)	(0.60-3.43)	(0.87-13.50)	(0.80-13.26)
Never	REF	REF	REF	REF	REF	REF

**Team sport participation**						
Yes, participating	1.20	1.69	1.07	0.95	2.19	1.49
	(0.40-3.58)	(0.52-5.52)	(0.35-3.29)	(0.29-3.15)	(0.48-10.02)	(0.30-7.43)
Quit	0.55	0.64	1.50	1.42	2.75	2.16
	(0.17-1.73)	(0.20-2.08)	(0.64-3.53)	(0.58-3.48)	(0.73-10.38)	(0.55-8.53)
Never	REF	REF	REF	REF	REF	REF

**Sport success/result** ^**cont**^	1.84	1.84	1.22	1.15	2.02	1.84
	(1.09-3.37)	(1.02-3.32)	(0.73-2.02)	(0.67-1.97)	(1.05-3.91)	(0.95-3.73)

**Experience in sport** ^**cont**^	1.34	1.60	1.64	1.70	1.92	1.78
	(0.88-2.05)	(1.01-2.53)	(1.21-2.41)	(1.12-2.58)	(1.14-3.24)	(1.02-3.12)

**Number of training sessions per week ** ^**cont**, **A**^	1.21	1.50	1.21	1.23	1.79	1.61
	(0.76-1.94)	(0.88-2.57)	(0.85-1.76)	(0.81-1.87)	(1.07-3.01)	(0.91-2.85)

*∗* REF, referent value, Crude, nonadjusted logistic regression, Adjusted, logistic regression adjusted for age, gender, socioeconomic status, and conflict with parents, ^cont^, denotes variables observed as continuous for the purpose of logistic regression calculations, ^A^, variable where only former and current athletes were observed, and ^*¥*^, participants who were users of illicit drugs at study baseline were not included in this analysis.

## Data Availability

The data file is provided as supplementary file of the submission and is also available here: https://www.dropbox.com/s/q57xqqn5u5khbg4/data%20file.xlsx?dl=0.

## Supplementary Materials

Supplementary Materials Supplementary material: (a) questionnaire form used in the study (translated from local language). (b) Parental consent for the participation in the study for their child/children (translated from local language). (c) Data file. (d) Supplementary table: attrition bias analysis.
