# Supplementary material for: Are Sports-Related Factors Correlated to the Prevalence and Initiation of Illicit Drug Misuse in Adolescence? Prospective Study in Older Adolescents
Source: Biomed Res Int. 2018 Nov 28;2018:1236284. doi: 10.1155/2018/1236284 (PMC6304193; doi:10.1155/2018/1236284)
Supplement: Supplementary Materials — Supplementary material: (a) questionnaire form used in the study (translated from local language). (b) Parental consent for the participation in the study for their child/children (translated from local language). (c) Data file. (d) Supplementary table: attrition bias analysis. [file 1236284.f1.zip › Supplementary Materials/PARENTAL consent form.docx]

**PARENTAL/GUARDIAN CONSENT FORM (translated from local language)**

Dear Parents/Guardians

In cooperation with two Universities (University of Mostar – Bosnia and Herzegovina, and University of Split - Croatia), school authorities perform a study on sport factors and illicit drugs consumption among children in our school (name of the study Drugs and correlates – DC). The main idea is to evaluate the: (i) level of sport participation, and (ii) consumption of illicit drugs in our students your children. The project will be performed by anonymous questionnaires (attached as a supplement to this letter). Students will be tested at the beginning of 3^rd^ year of high school, and again – at the end of 4^th^ year of high school. Study is anonymous, and as you can see, children will not be asked for personal details which could connect them to provided answers. However, since we intend to observe the changes in tested variables during the study-period, they will be asked to use self-selected confidential code. Again, even this code will remain confidential since no one will ask them to specify it in any circumstance.

At the beginning of the study (i.e. beginning of the 3^rd^ year of high school) all children are minors. Therefore, we must ask for parental/guardian consent for study participation. In order to provide your consent for the participation of your child in the study, please fill the form bellow

The participation is absolutely voluntary and if you (or your child) have any doubt about it please feel free to ignore this invitation. Also, if you need more information please feel free to contact us at cell phone number XYXYXYXYXYXY and or by e-mail: XYXYXYXYXYXYXYX.

Thank you in advance!

I give permission for my child ________________________________ to take part in the investigation explained above

PARENT’S/GUARDIAN’S NAME (IN CAPITAL LETTERS) PARENT’S/GUARDIAN’S SIGNATURE

________________________________________ _____________________________
