# Supplementary material for: Are Sports-Related Factors Correlated to the Prevalence and Initiation of Illicit Drug Misuse in Adolescence? Prospective Study in Older Adolescents
Source: Biomed Res Int. 2018 Nov 28;2018:1236284. doi: 10.1155/2018/1236284 (PMC6304193; doi:10.1155/2018/1236284)
Supplement: Supplementary Materials — Supplementary material: (a) questionnaire form used in the study (translated from local language). (b) Parental consent for the participation in the study for their child/children (translated from local language). (c) Data file. (d) Supplementary table: attrition bias analysis. [file 1236284.f1.zip › Supplementary Materials/Questionnaire form.docx]

***Questionnaire form (translated from local language)***

**THREE DIGIT CODE (self-selected)**: __________________

**Age**: __________________________

**Gender**: M F

**Individual sport participation:**

- 1. Yes, still participating
  2. Quit
  3. Never

**Team sport participation:**

1. Yes, still participating
2. Quit
3. Never

**Highest sport success/result achieved**

1. Never competed
2. Local rank
3. National rank
4. International rank

**Experience in sports**

1. Never been involved
2. < 1 year
3. 2-5 years
4. 5 years

**Number of training sessions per week (skip the question if never been involved in sports)**

1. 1-2 per week
2. 3-5 per week
3. Every day
4. Sometimes even 2 sessions per day

**Socioeconomic status (of your family)**

1. Under average
2. Average
3. Above average

**How often are you in conflict with your parents/guardians?**

1. Never
2. Rarely
3. From time to time
4. Frequently

**How often did you try following substances? (Please provide the answer with an “X” for each substance)**

|  | **Never** | **1-2 times** | **3-5 times** | **6-9 times** | **10-19 times** | **20-39 times** | **40+ times** |
| --- | --- | --- | --- | --- | --- | --- | --- |
| Ephedrine |  |  |  |  |  |  |  |
| Cocaine |  |  |  |  |  |  |  |
| Speed |  |  |  |  |  |  |  |
| Ecstasy |  |  |  |  |  |  |  |
| Marihuana |  |  |  |  |  |  |  |
| Hashish |  |  |  |  |  |  |  |
| LSD |  |  |  |  |  |  |  |
| Heroin |  |  |  |  |  |  |  |
| Ketamine |  |  |  |  |  |  |  |
| GHB |  |  |  |  |  |  |  |
| Sedatives (*Valium*, Rohypnol, Vicodin, etc.) |  |  |  |  |  |  |  |
| Inhalants (*glue, nitro, paints, etc.*) |  |  |  |  |  |  |  |
