# Supplementary material for: Are Sports-Related Factors Correlated to the Prevalence and Initiation of Illicit Drug Misuse in Adolescence? Prospective Study in Older Adolescents
Source: Biomed Res Int. 2018 Nov 28;2018:1236284. doi: 10.1155/2018/1236284 (PMC6304193; doi:10.1155/2018/1236284)
Supplement: Supplementary Materials — Supplementary material: (a) questionnaire form used in the study (translated from local language). (b) Parental consent for the participation in the study for their child/children (translated from local language). (c) Data file. (d) Supplementary table: attrition bias analysis. [file 1236284.f1.zip › Supplementary Materials/Supplementary Table 1.docx]

Supplementary Table 1

Responders vs. Non-responders’ analysis of attrition bias (Chi Square test), for gender and initial status of illicit drug misuse (IDM)

|  | Responders | Non-responders | Chi square (p) |
| --- | --- | --- | --- |
| Illicit drug misusers | 19 | 3 | 2.51 |
| Illicit drug non-users | 413 | 24 | (0.11) |
|  |  |  |  |
| Males | 230 | 21 | 6.17 |
| Females | 202 | 6 | (0.01) |
